# Decrease in reproductive desires among non-parent heterosexual women during the COVID-19 pandemic in Poland: the role of epidemiological stress, socioeconomic status, and reproductive rights

**DOI:** 10.3389/fpubh.2025.1462215

**Published:** 2025-03-05

**Authors:** Urszula M. Marcinkowska, Krzysztof K. Kasparek, Michał Zabdyr-Jamróz, Marta Kozłowska, Ilona Nenko

**Affiliations:** ^1^Department of Environmental Studies, Faculty of Health Sciences, Jagiellonian University Medical College, Krakow, Poland; ^2^Centre for Evaluation and Analysis of Public Policies, Jagiellonian University, Krakow, Poland; ^3^Department of Health Policy and Management, Faculty of Health Sciences, Jagiellonian University Medical College, Krakow, Poland; ^4^Mercator Forum Migration and Democracy, Institute of Political Science, TU Dresden, Dresden, Germany

**Keywords:** fertility, intentions, reproduction, children, plans, crisis

## Abstract

**Introduction:**

Deterioration of economic conditions, societal uncertainty, and negative expectations about the future have all been linked to delayed childbearing plans. All these negative circumstances can be related to epidemiological stress, which in turn becomes one of the culprits for changes in fertility plans. This study aims to analyze the individual factors that decrease the probability of wanting to have children after exposure to epidemiological stress from the coronavirus disease 2019 (COVID-19) pandemic.

**Methods:**

Recruitment was conducted between April and July 2021. Participants who were heterosexual, non-parent, and non-pregnant without a diagnosis of infertility completed an online, anonymous survey providing information on sociodemographic variables, COVID-19 exposure, COVID-19-related stress, and changes in their reproductive desires. Multiple logistic regression models were used to analyze the data. Participants were also given the opportunity to provide a descriptive explanation for changes in fertility desires due to the pandemic or the political situation (abortion restrictions coinciding with the pandemic in Poland), which was then used for qualitative analysis.

**Results:**

A total of 706 participants completed the survey (mean age = 28.11, SD = 4.87, min = 19, max = 47). We found that (1) the desire to have children decreased in 43.3% of respondents, and (2) women with higher levels of epidemiological stress were more likely to report a decrease in their desired number of children than the less-stressed ones, after adjusting for potential covariates (aOR = 1.064, 95%CI = 1.03–1.10, *p* < 0.001). Disease exposure yielded no significant results (aOR = 0.862, 95% CI = 0.73–1.02, *p* = 0.072). Additionally, 70% of participants declared a decrease in their willingness to have children due to the political situation. All models were adjusted for age, education, place of residence, socioeconomic and relationship status.

**Conclusion:**

The situation in Poland during the COVID-19 restrictions provided a unique combination of political and epidemiological stressors, showing that women’s reproductive desires were related to pandemic stress (less so with the exposure to disease) and limitation of reproductive rights.

## 1 Introduction

Family dynamics are expected to change in response to a crisis. Deterioration of economic conditions that follow crises, societal uncertainties, and negative expectations about the future have all been linked to delayed reproductive intentions (1–5). Multiple studies from industrialized countries indeed showed a drop in fertility rates between 2019, 2020, and 2021 (6, 7), co-occurring with the introduction of the pandemic-related restrictions globally. The magnitude of the decrease varied (usually between 3 and 20%), with some countries, like Denmark, Finland, or Norway, not experiencing the drop at all. In addition, the effects of the pandemic might differ between men and women (8). It has been found that women have more frequently experienced greater challenges than men when trying to balance work and family responsibilities during the pandemic (9). This might have in turn increased the pandemic-related stress in women more than in men, and as a result, the observed change in fertility plans would be more pronounced in women than in men (10).

It is also important to distinguish between measuring solely the fertility rate decrease and the decrease in one’s willingness to have children. Depending on the socioeconomic and relationship status, health, and contraception or *in vitro* fertilization (IVF) accessibility, the correlation between one’s willingness to have children and the actual number of children one will have can vary. Nevertheless, a decrease in the willingness to have children is surely one of the key factors contributing to lower fertility rates. Studies from the United States showed that 34% of surveyed participants expressed a desire to delay childbirth or to have fewer children due to the pandemic (11). A study conducted in Italy found that 37.3% of adults who had planned to have a child before the pandemic abandoned that intention (12). The factors contributing to the decrease in fertility rates can be derived from both societal and individual levels, and they are numerous, complex, and hard to untangle.

Variables determining the reproductive plans of non-parent individuals are manifold. However, socioeconomic factors appear to play a significant role in reproductive behaviors. People postpone the birth of their children (both the first and the subsequent ones) when uncertainty about economic and material security is high (13, 14). Another study from Germany (15) indicated that precarious employment delays family formation in a similar way among non-parent women and men. The study indicates that this effect correlated with more restrictive policies concerning unemployment benefits and the growing necessity for supplementing family income with a second job. Other factors related to fertility plans can be stress (caused by both sanitary restrictions and fear of illness) and exposure to possibly life-threatening diseases. Similarly to other pronounced crises such as natural disasters (16, 17) or acts of terror (18), we could expect that a global pandemic will cause a substantial decrease in mental health quality and an increase in overall stress among most individuals. The prevalence of post-traumatic stress and anxiety after exposure to mass crises is well documented (19). Since stressful and unstable life situations have been shown to impact fertility plans (20), we would expect to see a greater decrease in fertility motivation among women whose stress and exposure to disease were greater.

Owing to the unexpected confounding event in Poland, the aims of this study were amended. The ruling of the Constitutional Tribunal removed the exception in anti-abortion law allowing for termination in case of fetus malformation in October 2020. This narrowed the women’s reproductive rights in Poland during the pandemic. The ruling sparked a massive protest movement. In the spring, when our study was conducted, the issue remained prominent in the media as the first legal proceedings against those arrested during the protests began. As a result, the events were highly salient. We therefore decided to include control questions asking about the influence of the political situation on reproductive desires and gave the respondents an opportunity to write an open answer to them.

In this study, we aimed to analyze which individual factors were related to changes in fertility desires after exposure to epidemiological stress in the form of the coronavirus disease 2019 (COVID-19) pandemic. We analyzed how the intentions of non-parent women to have fewer children were related to sociodemographic factors. Two variables were related to epidemiological stress: disease exposure and pandemic-related stress. We hypothesized that women who had higher disease exposure and pandemic-related stress more frequently decreased their desired number of children. We also expected that socioeconomic status would play a role in this relationship—women with better socioeconomic status would be less affected by epidemiological stress.

## 2 Materials and methods

The study was conducted according to ethical standards as described in the Declaration of Helsinki and the Council for International Organizations of Medical Sciences (CIOMS) 2016 guidelines. The Ethics Committee opinion was received on 25 November 2020 from The Jagiellonian University Ethics Committee under the number 1072.6120325.2020, and consent was obtained from all participants.

### 2.1 Survey

An online survey was posted on the Qualtrics Platform and was promoted in online groups and social media between April and July 2021 (pandemic-related restrictions were introduced in Poland in March 2020). The survey followed the guidelines of the Checklist for Reporting Results of Internet E-Surveys (CHERRIES) (21). The online survey was pre-tested before the opening of the recruitment by randomly selected individuals (personal communication from the authors of the study), and the Ethical Review Board opinion was secured before the initiation of the recruitment (see above). A convenience sample was recruited through an open survey (no password protection) promoted on social media (both the authors’ and the university’s accounts, including paid promotion on social media) and on websites related to reproductive health. Informed consent was secured by clicking on the right option (the survey was not mandatory, participants could stop at any time, and could return to the previously answered questions). No personal data protection measures were in place due to the anonymous nature of the survey, and there was no time limit for completion of the survey. Internet Protocol (IP) address check was used to prevent multiple entries (no IP information was stored though). Response and completion rates were not tracked, as entries with missing data were not included in the analyses.

In the first section, we collected data on the sociodemographic characteristics of the participants, including age, education, place of residence, financial situation, nationality, number of children, relationship status, diagnosed infertility, pregnancy, and sexual orientation.

The second section focused on COVID-19-related questions: whether participants had tested positive for severe acute respiratory syndrome coronavirus 2 (SARS-CoV-2), whether their family or acquaintances had tested positive, or whether they had any personal contact with someone infected with SARS-CoV-2. Responses to these four questions were coded 0 (no) or 1 (yes) and summed in the form of a COVID-19 exposure index (min = 0, max = 4). For the second set of pandemic-related questions, we used the International Adjustment Disorder Questionnaire (IADQ) survey (22), adjusted for the COVID-19 occurrence (23), with responses in the form of a 5-point Likert scale, where the higher number indicated the highest level of stress (min = 6, max = 30).

The third section consisted of questions about fertility plans. All the women were asked whether the COVID-19 pandemic has changed their reproductive plans, as well as how many children they wanted to have before the pandemic and now (i.e., during the time of the survey, April–July 2021). The change of score in fertility desires was computed by subtracting the desired number of children before the pandemic from the desired number of children during the pandemic. We categorized the change in two forms: 0 = *remaining the same*, 1 = *declining*.

Control variables included age, partnership status, education, employment situation, self-reported financial situation, and place of residence. Age was used as a continuous variable in the analysis. The relationship status was coded in the following way: 0 = *single* (included participants who were single or not in stable relationships) and 1 = *in a stable relationship* (including marriage). Education was coded as 0 = *no university degree* (comprised participants with high school or lower level of education), 1 = *university degree* (bachelor’s, master’s degree, or PhD). Participants’ financial situation was assessed with the question, “How would you describe your financial situation?” offering five response options, ranging from very bad to very good. Owing to the small number of participants rating their financial situation as very bad or bad, the variable was coded in the following way: 0 = *average of lower* (including very bad/bad/average), 1 = *good or very good*. Finally, participants reported their place of residence as one of the following categories: 1 = *village*, 2 = city <200 k residents, and 3 = city 200 k + residents. Furthermore, we decided to add a question related to the political situation, as during the COVID-19 pandemic, reproductive rights regarding access to abortion were limited in Poland. The variable “Does the political situation in Poland impact your reproductive plans?” was answered with the following options: “No impact,” “Impact: It makes me want to have fewer children,” “Impact: It makes me want to have more children,” and “Other.” Women could then proceed to answer an open-ended question to describe in what way the political situation impacted their reproductive plans.

### 2.2 Participants, inclusion criteria, and confounding variables

Women diagnosed with infertility or below 18 years of age were not included in the survey. In the final sample included in the analyses, only eight women reported an increase in the desired number of children. Although interesting as to where the increase stems from, we did not include these participants in the current analyses due to the inability to compare such a small group (1% of the total sample) to the two other groups of interest (decrease and no change in reproductive intentions). In the final sample, there were no women who would declare that the current political situation increased their desire to have children.

As the aim of the study was to determine what factors are related to the change in the desired number of children, in the final analyses, we included only participants who declared before the pandemic that they actually did want to have progeny.

### 2.3 Statistical analysis

To test how the selected independent variables change the odds of wanting fewer children, a logistic regression model was employed. To assess model fit, the Hosmer–Lemeshow test and McFadden pseudo-R2 statistic were calculated. We decided to include age, education, self-perception of participant’s financial status, and place of residence as confounding variables. For the pandemic-related scores (stress and exposure), two indices were created, and their effectiveness was measured by the *α* reliability coefficients. Additionally, we included the question on the political situation as we expected it could be of importance. All analyses were conducted using the Stata 17 software (command “logit”). The statistical significance level was set at *p* < 0.05.

### 2.4 Qualitative analysis of facultative open questions

The participants were given an opportunity to answer two open-ended questions: one about the pandemic and one about the political situation in the country. We conducted a qualitative text analysis of 109 responses from women who stated that their willingness to have children had decreased. As the answers were short and concise, no thick analyses – i.e. an elaborated in-depth method (24) – were applicable; instead, we applied literal readings (25). Furthermore, indexing was used to build categories. As the pandemic was given a full battery of questions elsewhere, we focused here on the salience of the anti-abortion law and the political atmosphere in the country. We coded whether the respondents mentioned abortion directly, mentioned it indirectly (e.g., “the ruling of the Tribunal”), insinuated (e.g., “what this government does to women”), or not mention it at all. The stated reasons were indexed and grouped into five sociopolitical categories, as well as one personal category, which we labeled “ecological-ethical” (Table 1). The indexing was executed by two coders in a joint procedure (MK and MZJ), and the codes were applied when both coders agreed. The codes were not exclusive; multiple codes could be applied to a single passage.

**Table 1:** Indexing of answers to open-ended questions about the impact of the political situation on reproductive desires (*N* = 109).

Limiting access to abortion as a deterrent to having children in Poland:	*N* (%)
Directly stated	36 (33%)
Indirectly stated or insinuated	24 (22%)
Not stated	49 (45%)
Other political and systemic issues deterring from having children in Poland:^a^	
Poor conditions in the pregnancy and birth-related healthcare system	43 (39%)
General lack of trust in the state institutions and the government	36 (33%)
Poor state support and care systems (schools, labor market, support for people with disabilities and for parents)	14 (13%)
Gender inequality in Poland, limiting or infringing on women’s rights	12 (11%)
Other	10 (9%)
Too high or misdirected redistribution	1 (1%)
Personal ecological-ethnical reasons	
Mentioned	6 (5.5%)
Not mentioned	103 (94.5%)

a Multiple answers possible.

## 3 Results

From the total sample of 1,539 non-parent women who completed the survey, we excluded 833 of them from the analysis, based on the following circumstances: declaration of non-heterosexual orientation (*n* = 194), no desire to have any children before the start of the COVID-19 pandemic (*n* = 455), being currently pregnant (*n* = 99), and incomplete records in the variables needed for the analysis (*n* = 85). The final sample comprised 706 women aged 28.1 years on average (SD = 4.9, min = 19, max = 47). The majority of participants had higher education (77.2%), lived in larger cities (over 200 k population) (62.5%), and were in a stable romantic relationship (83.8%). Women declared that before the beginning of the pandemic, they planned to have two children on average. When asked about the number of children they wanted at the current moment, the median number of wanted children was still two, but 20.2% of women declared they did not want to have any children at the moment. Descriptive statistics can be found in Table 2.

**Table 2:** Sample descriptive statistics of the studied group of women (*N* = 706). The demographic characteristics were summarized as the mean with standard deviation (SD), and range for continuous variables, and as frequency counts (percentages) for the categorical variables.

Variables	Mean (SD), range/n (%)
Age (years)	28.1 (4.9), 19–47
Number of children wanted before the pandemic	
1	180 (25.5%)
2	304 (43.1%)
3	184 (26.1%)
4	28 (4.0%)
5	5 (0.7%)
6 or more	5 (0.7%)
Number of children desired at the time of data collection	
0	143 (20.2%)
1	201 (28.5%)
2	232 (32.9%)
3	110 (15.6%)
4	14 (2.0%)
5	3 (0.4%)
6 or more	3 (0.4%)
Relationship status	
Single	114 (16.2%)
In a stable relationship	592 (83.8%)
Education	
No university degree (high school or lower level of education)	161 (22.8%)
University degree (bachelor’s degree, master’s degree, PhD)	545 (77.2%)
Own financial situation	
Average or lower	300 (42.5%)
Good or very good	406 (57.5%)
Place of residence	
Village	100 (14.2%)
City up to 200,000 residents	165 (23.3%)
City above 200,000 residents	441 (62.5%)
Change in the desired number of children because of the pandemic	
Remained the same	400 (56.7%)
Decreased	306 (43.3%)
*COVID-19 related stress, Mean (SD), range 6–30*	15.34 (5.68)
COVID-19 exposure	
Contact with a person with the SARS-CoV-2 virus, yes	436 (61.8%)
Family members with the SARS-CoV-2 virus, yes	506 (71.8%)
Friends or acquaintances with the SARS-CoV-2 virus, yes	654 (92.6%)
Own SARS-CoV-2 virus test result, yes	102 (14.5%)
*COVID-19 exposure index, Mean (SD), range 0–4*	2.41 (1.01)
Does the political situation in Poland impact your reproductive plans	
No impact	167 (23.7%)
Impact – it makes me want to have fewer children	496 (70.2%)
Impact – It makes me want to have more children	8 (1.1%)
Other	35 (5.0%)

The pandemic-related indices yielded moderate (*α* = 0.52) and satisfying (α = 0.89) levels of reliability coefficient for exposure and stress, respectively. Of all the women who wanted to have children before the pandemic, 43.3% declared to want fewer children at the time of the survey. Each additional point on the COVID-19-related stress scale (6–30) increased the odds of wanting fewer children by 6% after adjusting for potential covariates (aOR = 1.06, 95%CI = 1.03–1.10, *p* = <0.001, Table 3). Women more stressed by the pandemic were more likely to report a decrease in the number of children they desired. However, the relationship between COVID-19 exposure and the planned number of children was not statistically significant (aOR = 0.86, 95%CI = 0.73–1.02, *p* = 0.076).

**Table 3:** Logistic regression model testing the change in the desired number of children (0 = remaining the same, 1 = declining).

	aOR	S.E.	*z*	*p*	95% CI
*COVID-19 exposure index*	0.86	0.07	−1.77	0.076	0.73–1.02
*COVID-19 related stress*	1.06	0.02	4.01	<0.001	1.03–1.10
*Age (years)*	1.05	0.02	2.46	0.014	1.01–1.10
*Place of residence*					
Village	Reference				
City up to 200,000 residents	0.76	0.22	−0.96	0.336	0.43–1.34
City above 200,000 residents	0.69	0.18	−1.41	0.158	0.41–1.15
*Education*					
No university degree	Reference				
University degree	0.79	0.19	−0.98	0.325	0.49–1.27
*Relationship status*					
Single	Reference				
In a stable relationship	0.71	0.17	−1.42	0.155	0.44–1.14
*Own financial situation*					
Average or lower	Reference				
Good or very good	0.64	0.11	−2.51	0.012	0.46–0.91
*Political situation*					
No impact	Reference				
Makes me want children less	6.45	1.57	7.65	<0.001	4.00–10.40
Makes me want children more	0.72	0.46	−0.55	0.582	0.23–2.29
Other	1.80	1.58	0.67	0.501	0.32–10.03
*Constant*	0.07	0.04	−4.15	<0.001	0.02–0.24

Among the tested demographic confounders, age was significantly related to the desired number of children—older women were more likely to report a decrease in the desired number of children than younger ones (aOR = 1.05, 95%CI = 1.01–1.10, *p* = 0.014, Table 3). In addition, the financial situation was related to the decrease—women who judged their financial situation as good or very good were less likely to report a decrease in the desired number of children (aOR = 0.644, 95%CI = 0.46–0.91, *p* = 0.012, Table 3). Place of residence and education were not significantly associated with the decrease in the desired number of children.

**Figure 1:**
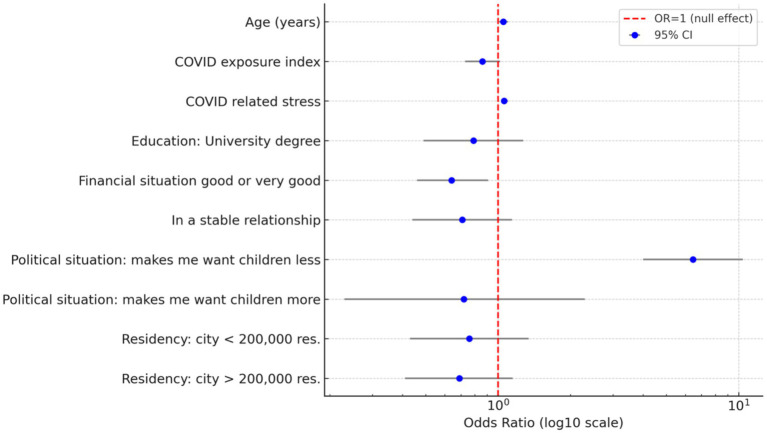
Coefficients plot for the change in the desired number of children (0 = remaining the same, 1 = declining); blue dots – OR, horizontal bars – 95%CI, and red dashed line OR = 1 (for reference groups see Table 3).

Women who declared a negative impact of the Polish political situation on their reproductive plans were over six times more likely to decrease the number of wanted children in comparison to those who did not report any impact (aOR = 6.45, 95% CI = 4.00–10.40, *p* < 0.001, Table 3). The results were stable and fitted the data (R^2^_McFadden_ = 0.15, Chi^2^_(11)_ = 146,77, *p* < 0.001, H-L_chi_^2^_(8)_ = 5.24, *p* = 0.732). See Figure 1 for a coefficient plot for all included variables.

The majority of women in our sample stated that the political situation in the country negatively impacted their reproductive plans. The majority of women who answered the open-ended questions (55%) mentioned access to abortion as discouraging to have children either directly or indirectly, or insinuated it as a reason without directly calling it so (Table 1).

## 4 Discussion

Overall, we found that in 43.3% of non-parent women, the desired number of children decreased. The reduction in the number of children women desired was related to pandemic stress, financial situation, age, and political situation. This result is comparable to a previously published study from Shanghai (26), where authors found that 3 in 10 couples decided not to have children after the COVID-19 outbreak. Our result may be higher because our sample included women who reported a decreased number of desired children—both those who entirely gave up on having children and those who still wanted children, but fewer. Our study also complements the results obtained by another team based on a Polish representative sample, which found that 20% of respondents reported postponing or foregoing their reproductive intention during or after the COVID-19 pandemic (27). Another study based on an Austrian sample reported that the intended family size was not changed during the pandemic. Only 7.6% of participants reported a decreased desire to have children due to the pandemic (however, the study was based on both parents and non-parents, as well as men and women) (28). Another study from Italy highlighted the importance of occupational and financial factors in shaping the impact of COVID-19 on fertility intentions (29). As some studies mention, “contrary to some initial expectations,” the coronavirus pandemic did not bring a lasting “baby bust” in most of the analyzed countries (30). Overall, based on the results from multiple cross-cultural samples, the effects of the pandemic on fertility desires remain incoherent (31).

### 4.1 Reproductive plans and reproductive rights

Pandemic stress was related to the reproductive plans—women who were more stressed (scoring higher on the COVID-19-related stress index) more frequently reported a decrease in the desired number of children compared to less-stressed women. These results are in line with previously published studies showing that life instability (an inevitable result of the pandemic) is one of the most important factors influencing fertility desires (20). The relation between the decrease in the number of desired children and disease exposure (measured by the COVID-19 exposure index) did not reach statistical significance.

Our results point to the importance of psychological factors on reproductive intentions in line with previous studies. It has been shown that when the stress level rises due to external conditions, reproductive plans are affected. For example, in one recent study, fertility plans were negatively correlated with the perceived importance of climate change (32). Similarly to our results, a recent study based on a Polish representative sample identified a lowered sense of financial security and worse mental wellbeing caused by the pandemic as the two main reasons for postponing childbearing (27). Another study showed the moderating effect that COVID-19 anxiety had on fertility intentions suggesting that through anxiety-reducing techniques, it is possible to increase childbearing desires (33). The role of psychological distress caused by the pandemic appears to be strongly scientifically grounded.

In addition, our results show a possible direction for targeted interventions. Improving women’s mental wellbeing by decreasing the perceived stress from epidemiological threats could lead to an increase in fertility intentions. Epidemiological stress was related to the decrease in the desired number of children, independent of sociodemographic factors. Simultaneously, the actual disease exposure was not significantly related to reproductive intentions. Interventions designed to decrease epidemiological stress and anxiety could then have a greater impact on women’s reproductive intentions than the actual epidemiological situation.

Additionally, research has shown that higher risk aversion in women decreases their likelihood of becoming a parent (15). Hypothetically, this mechanism could mediate the negative association between willingness to have children and epidemiological stress. Women who fear the disease more may have higher risk aversion, making them more prone to decrease the desired number of children under epidemiological stress.

### 4.2 Reproductive plans and financial status and age

Unsurprisingly, lower self-perceived financial status was related to a decrease in the number of desired children. In other words, a worse financial situation increased the odds of wanting fewer children among non-parent women, even to the extent of discouraging them from having children altogether. This corresponds to a previous study (15) indicating that precarious employment delays family formation among both genders. Additionally, other studies have also shown that perceived economic status significantly contributes to the postponement of having children in wealthy countries (13).

Older women were more likely to decrease their desired number of children than younger women. Previous studies have shown that older non-parent women frequently have more “childfree ideals” than younger non-parent women, possibly because of “the downward adjustments in the ideal number of children among those who remained childless involuntary” (34). The more frequent decrease in the desired number of children among older women could also stem from the fact that older women desired greater numbers of children before the pandemic (hence, for the younger ones, the decline is limited by the “floor effect”) (35). However, this may also be the result of negative assessment of childbearing opportunities, as late pregnancies are more risky in general and also have a higher percentage of fetus malformation—and older women decide not to risk such pregnancy without adequate medical support.

### 4.3 Reproductive plans and political situation

Our results show that the political situation was of great significance: over 70% of participants declared that it made them want to have fewer children. An important factor in analyzing changes in reproductive plans in Poland during the pandemic is the coincidental, abrupt restriction of reproductive rights, specifically access to abortion following the October 2020 ruling by the Polish Constitutional Tribunal (36). The ruling was announced approximately half a year after the first COVID-19 restrictions and sparked mass protests. Conscious of the possible negative impact of abortion rights restrictions on maternal health (37), as well as on children’s socioeconomic prospects (38, 39) and, consequently on the reproductive intentions of potential parents, we included a question in the survey on the self-judged importance of the political situation on participants’ reproductive plans. In fact, a vast majority of women reported that the political situation was related to their reproductive plans. Moreover, including the perceived political situation in the statistical model yielded significant results: women who reported that the political situation influenced them also reported a strong decrease in desire to have children.

However, it is important to note (see also *Limitations* section) that due to the broad nature of the question, we were unable to identify the specific aspects of the political situation that influenced participants’ responses. An additional qualitative analysis of answers (*N* = 109) to facultative open-ended questions indicated that the ruling on abortion contributed significantly to this result, as it was mentioned by more than half of the participants. The analysis further indicated the strong feelings of uncertainty about safety or even survival in case of pregnancy complications as a likely driver of women’s unwillingness to reproduce in such circumstances. What supports the hypothesis that respondents interpreted “political situations” as the political upheaval in access to reproductive rights are public opinion polls conducted in Poland after the Constitutional Tribunal’s restriction of abortion access. In these polls, 57% of women in 2021 and 67% in 2022 believed that “the [Tribunal’s] ruling resulted in less willingness to have children” (40). Nevertheless, a separate, in-depth analysis concerning the impact of the political situation is needed to attain more firm conclusions.

Overall, our results suggest formulating a cautious hypothesis, to be tested in future studies, that under certain conditions, reducing access to abortion, somewhat paradoxically, might result in diminishing the fertility rate due to reduced willingness of women to have children caused by fears concerning reproductive health and sense of threatened autonomy. This is a relevant consideration adding to already noted concerns over side effects of abortion restrictions [Cf. e.g. (41)]. It is especially concerning for countries (such as Poland), with low fertility rates that may be interested in pro-natalist policies, as their effects could be negated by further abortion bans.

## 5 Limitations and strengths

There are some limitations of the current study, which we list along with a description of their significance and how they were, or could be, accounted for. First, the sample was not representative of the Polish population. The women who decided to participate in the survey were typically from higher education backgrounds and from larger cities (22% of participants did not have a bachelor’s degree and 14% lived in a village). This should be considered when extrapolating current results to the general population. Nevertheless, the sample size is notable (*N* = 706), and within the sample, participants showed a great degree of variation in the number of children they wanted to have, in COVID-19 stress and exposure, and in socioeconomic status. Additionally, the general result of approximately 40% of women reporting a decrease in fertility intentions is comparable to previously published results. Nevertheless, we advise caution when interpreting the results to the general population, as the current results were obtained based on a sub-sample of Polish women who were highly educated and resided mostly in bigger cities. Further research based on a representative sample is needed to understand how the same factors operate in rural areas.

Second, the cross-sectional design of the study did not indicate the temporal and causal relationships between the variables, which also limits the interpretation of the findings. To account for this issue, future studies should implement a longitudinal design, where one participant is being monitored for an extensive period of time, and their changes in reproductive plans are being reported multiple times together with particular events in their life during that time. Next, it is possible that the measure we chose for estimating COVID-19 exposure did not depict sufficiently the real situation; that is, the measure did not account for the size of the social network and family network the respondents had. Nevertheless, we believe that the narrow set of questions we have used for estimating COVID-19 exposure was sufficient to provide a crude estimate of the participants’ COVID-19 prevalence in close social surroundings. Furthermore, as women reported very high exposure indices, we believe it would have been very difficult for them to report the exact numbers of friends and family members who tested positive—and that in turn would inflate the measurement error.

Additionally, all self-reported information from online surveys includes a certain level of measurement error based on participant’s willingness to answer honestly, to please researchers—social desirability (less probable in an online, anonymous study), but also their inability to recall correctly their own state—recall bias (in our case, the number of children wanted before the pandemic and the introduction of the abortion restrictions). Furthermore, our open survey was conducted without an eligibility check; therefore, the self-selection of participants may have biased representativeness in unmeasured variables.

The *ad hoc* nature of the question on the impact of the political situation limited our ability to perform a more thorough investigation of the motives. The question itself is general and does not necessarily equate with abortion rights limitation. It should be noted that the general nature of the question combined with the context of mass protests possibly made the abortion issue answer as a default for “political reasons” or assumed, and other reasons had been added as compounding motives (55% of respondents directly or indirectly stated the abortion issue as the reason). We acknowledge these shortcomings. Nevertheless, although not within the scope of the current analysis, these results appear to be an important indicator of additional factors deterring women from having children and point to future areas of study.

## 6 Conclusion

Any decrease in reproductive intentions is especially worrisome, as in countries with low fertility (such as most WEIRD—Western, Educated, Industrialized, Rich, and Democratic—populations, including Poland), actual fertility rates are even lower than the ideal number for children individuals express for themselves (42). As among previously found reasons to postpone childbearing, uncertain life situations were one of the primary ones (and independent of sociodemographic situations) (20). We would expect that the pandemic stress and exposure to epidemiological threats would further decrease fertility intentions, independently from markers of low socioeconomic status. Our results support this hypothesis, but only partly. Overall, in 43.3% of non-parent women, the number of desired children decreased during the pandemic. The current analysis shows that older non-parent women with lower socioeconomic status and those who felt more pandemic-related stress were more likely to decrease the number of children they want in the future. Pandemic stress was related to the decrease in the desired number of children, but the actual disease exposure was not significantly related to reproductive intentions. Moreover, and in concurrence with earlier studies, the lower self-perceived financial status of non-parent women was related to the decrease in the number of desired children, even to the extent of discouraging them from having children at all. Interestingly, our results also showed that the political situation was significantly related to a reported decrease in the willingness to have children, including the limiting of reproductive rights of women.

Our results suggest that in order to have a positive impact on women’s reproductive intentions, public policy interventions should be aimed primarily at (1) decreasing epidemiological stress (2), addressing stress related to low socioeconomic status, and (3) addressing the uncertainty or disenfranchisement when it comes to reproductive rights.

## Data Availability

The raw data supporting the conclusions of this article will be made available by the authors, without undue reservation.
